# A Strange Case

**Published:** 1877-04

**Authors:** J. Clark Scott


### ARTICLE IX.

### *A Strange Case. Is There a Parallel?*

BY J. CLARK SCOTT, D. D. S.

On the 20th of July, 1875, Miss B———, of Columbus, Ohio, called at my office to consult me in regard to a wisdom tooth of the left side, inferior maxilla. I found that the tooth in question was just being erupted, and in consequence of the crowded condition the eruption was difficult. Abscess had already formed, and quite a quantity of pus was being discharged. I lanced the gum, made two injections, and instructed her to call the following day at nine a. m. She did so, and after another injection I dismissed the case, advising her to have the tooth extracted as soon as it was far enough through.

December 18th. Miss B. called again to consult in a case of dental disturbance, that for a greater part of a year had been causing her much suffering, beside much anxiety of mind, and made the following statement: "In the month of —— I went according to your advice to have the wisdom tooth extracted. The dentist made several unsuccessful attempts to remove it, and at last assured me that it was out, though he did not exhibit the tooth." Suffering and inflammation supervened rapidly, culminating some time afterwards in an abscess on the left side of the neck, which at this time presented an unsightly pit just behind and a little below the jaw. She was confined to her bed for ten weeks, not taking any nourishment except what could be drawn through the spaces between her teeth. The muscles of her face and neck became rigid and the jaws firmly locked. She was visited by Dr. J. A Dunn, of Columbus, and an examination made, which resulted in finding the empty socket of the *dens sapientia*, and by using the probe could discover a smooth round surface, which was undoubtedly the crown of the lost wisdom tooth. Dr. Dunn extracted the second molar, thinking by so doing he would be able to get at the dislodged tooth. But on account of the patient's condition he was unable to accomplish his object. Under treatment of Dr. Flowers (a physician) she (after long suffering) was able to leave her bed, and gradually grew better until at last she was able to attend to her duties. After this she consulted two or three surgeons who discovered a hard substance imbeded well down in the muscles below the left tonsil, and at the rear of the posterior fauces. Several attempts to remove the foreign body were unsuccessful. In passing my finger behind the velum palati, I distinctly felt a jagged and hard substance, the nature of which I could not determine. Upon the second insertion of the finger I had no great difficulty in descrying the offending occupant of so dangerous and near proximity to the epiglottis and the internal carotid artery, nor had I much doubt that it was the missing *dens sapientia.* Its position was a little to the left of the median line of the cervical column, and about three inches below the normal position in the inferior maxillary bone. It was seen with some difficulty, as the patient could not get her front teeth more than three-quarters of an inch apart, and there was more or less tumefaction of the tonsils. I first essayed its removal with small forceps, but failed for the want of light and space. Blood flowed more or less freely, which at one time reached the larynx, producing rather alarming conditions. The blood was however cleared away by violent coughing, and respiration restored. At one time it seemed my resources would fail me, but a happy expedient suggested. I took a lancet and cut the muscle upwards and to the left; after the bleeding had about subsided I took a steel instrument of about eight inches in length and curved it to suit the turns which would have to be made. I then passed the hook under the supposed tooth, gave it a lifting and forward force, when my anxieties were terminated by seeing the tooth thrown in front of the uvula upon the tongue, and as I threw the lady's head forward the long lost wisdom tooth fell into her hand. It is the bona fide left lower wisdom tooth. The crown is of average size and well formed. The fangs bifurcate, are short, and slightly curved at the apex. How did it get three inches down in the posterior fauces and become imbeded in the muscle? Was it pushed under the pterygoid muscle, and then gravitated? I have the tooth in my possesion, with the transverse alveolar substance between the roots.

N. B. This case is, to me at least, a very strange one, and I have never heard of but one other that in any way simulated it. This was a case that occurred in Detroit, Michigan but it was not attended with the extreme suffering and strange freaks as the one here reported.—*Dental Register.*

